# Value of diagnostic and therapeutic laparoscopy for patients with blunt abdominal trauma: A 10-year medical center experience

**DOI:** 10.1371/journal.pone.0193379

**Published:** 2018-02-22

**Authors:** Heng-Fu Lin, Ying-Da Chen, Shyr-Chyr Chen

**Affiliations:** 1 Department of Surgery, Far-Eastern Memorial Hospital, New Taipei, Taiwan, R.O.C; 2 Department of Emergency Medicine, National Taiwan University Hospital and National Taiwan University College of Medicine, Taipei, Taiwan, R.O.C; Campus Bio Medico University, ITALY

## Abstract

Laparoscopy has been used for the diagnosis and treatment for hemodynamically stable patients with penetrating abdominal trauma. This study evaluated whether diagnostic and therapeutic laparoscopy can be used as effectively in select patients with blunt abdominal trauma. All hemodynamically stable patients undergoing operations for blunt abdominal trauma over a 10-year period (2006–2015) at a tertiary medical center were included. Patients undergoing laparotomy were categorized as group A. Patients who underwent laparoscopy were categorized as group B. The clinical outcomes of the 2 groups were compared. There were 139 patients in group A and 126 patients in group B. Group A patients were more severely injured (mean injury severity score of 23.3 vs. 18.9, *P* < .001) and had a higher frequency of traumatic brain injuries (25.2% vs. 14.3%, *P* = .039). The sensitivity and specificity of diagnostic laparoscopy for patients in group B was 99.1% and 100.0%, respectively. No non-therapeutic laparotomies were performed in group B, and the success rate of therapeutic laparoscopy was 92.0% (103/112) for patients with significant intra-abdominal injuries. Patients in the 2 groups had similar perioperative and postoperative outcomes in terms of operation times, blood loss, blood transfusion requirements, mortality, and complications (all, *P* > .05). Laparoscopy is a feasible and safe tool for the diagnosis and treatment of hemodynamically stable patients with blunt abdominal trauma who require surgery.

## Introduction

In the era of minimally invasive surgery (MIS), laparoscopy is used in the management of abdominal trauma, but recommendations at the evidence level cannot be made because no randomized controlled trials have been available [1,2]. Laparoscopy has been shown to avoid the need for non-therapeutic laparotomy and shorten hospital stays for patients with penetrating abdominal trauma (PAT) [3], but controversies still exist with respect to the indications and therapeutic potential of laparoscopy [4]. Although concerns regarding missed injuries have been mentioned in initial reports [5,6], laparoscopy has gradually been accepted as an accurate tool for diagnosing patients with PAT in recent series as more experience with this procedure has accumulated [7,8]. Reports have also indicated that therapeutic laparoscopy can be successfully applied in select patients with varied intra-abdominal injuries, including those of the diaphragm, liver, spleen, and gastrointestinal tract [7,9].

For patients with blunt abdominal trauma (BAT), the role of laparoscopy is even less clear than for those with PAT due to relatively fewer reports [10]. One of the reasons for this is that more treatment options are available for patients with BAT. With advances in imaging and treatment, such as trans-arterial embolization (TAE) [11,12], non-operative management (NOM) has become the treatment of choice for most hemodynamically stable patients with BAT. On the other hand, emergency laparotomy can be life saving for patients in shock and unresponsive to fluid resuscitation. Laparoscopy, however, could be beneficial in some select situations, such as for patients with isolated intra-abdominal fluid accumulation of uncertain origin shown on computed tomography (CT) scans [13]. There are reports of the use of laparoscopy for blunt bowel perforations [14,15], liver injuries [13,16], and spleen injuries [17,18]. Our previous studies suggest the benefits of laparoscopy in avoiding the need of laparotomy for select patients with BAT, except for those with spleen injuries [13,15]. However, the actual role of therapeutic laparoscopy for patients with BAT remains undefined because the study focused on select indications in a limited number of patients and compared patients using an open approach on the basis of historical cohorts.

As experience in the use of laparoscopy for abdominal trauma accumulates, the application of laparoscopy for BAT patients has been expanded at our institution. The purpose of this study was to evaluate the effectiveness of laparoscopy in hemodynamically stable patients with BAT. We hypothesized that laparoscopy can be used safely in the diagnosis and treatment of select BAT patients with expanded indications by surgeons with adequate experience in advanced laparoscopic procedures and the use of laparoscopy for patients with PAT. We compared the outcomes of hemodynamically stable patients with BAT who underwent laparoscopy with those who underwent laparotomy over a 10-year period. All patients were treated at a single medical center by a single surgical team after adopting laparoscopy into the treatment algorithm for BAT patients.

## Materials and methods

### Patients

This study protocol was approved by the Institutional Review Board, and because of its retrospective nature, the requirement of informed patient consent was waived. We retrospectively reviewed the medical records of all patients with BAT from the trauma registry database at a single center, where trauma surgeons were responsible for trauma and acute care surgeries. These patients were evaluated over a 10-year period from January 1, 2006 through December 31, 2015. At our institution, laparoscopy has been incorporated into the treatment algorithm for hemodynamically stable patients with abdominal stab wounds since 2003 and for select patients with BAT since 2006. Patients included in the analysis who underwent laparotomy for BAT were categorized as group A, and those who underwent laparoscopy were categorized as group B.

Demographic and clinical data were recorded, including vital signs in the emergency department (ED), abbreviated injury scale (AIS) abdominal scores, injury severity scores (ISSs), hemoglobin levels in the ED, associated injuries, associated traumatic brain injuries (TBIs), the use of TAE prior to surgery, indications for surgery, operative findings, therapeutic procedures performed, spleen salvage rates, rates of conversion to laparotomy, rates of non-therapeutic laparotomy, operation times, blood loss, length of hospital stay, length of intensive care unit (ICU) stay, hospital mortality, and postoperative complications. Complications of interest included missed injuries requiring reoperations, wound infections, intra-abdominal abscesses, and long-term complications, including intestinal obstructions and ventral hernias. First, we compared the outcomes of all patients who underwent surgical interventions in the 2 groups. Second, a subgroup analysis to compare the outcomes of patients who underwent operations for failed NOM of solid organ injuries (liver or spleen) was performed.

A laparoscopic procedure was considered negative if no injury could be identified. Laparoscopy was classified as therapeutic when surgical repair or resection of a significant intra-abdominal injury was performed and as non-therapeutic if surgical intervention had no impact on the patient’s outcome. Patients with conversion to an open procedure were included in the laparoscopy group in an analysis on an “intention-to-treat” basis. Laparotomy was similarly classified as either therapeutic or non-therapeutic.

#### Diagnostic and therapeutic laparoscopy

Patients eligible for laparoscopy were required to be hemodynamically stable (systolic blood pressure ≥ 90 mm Hg). After a primary survey according to Advanced Trauma Life Support (ATLS) principles, patients sustaining BAT with persistent hypotension and unresponsive to fluid resuscitation (systolic blood pressure < 90 mm Hg) were taken directly to the operating room for an exploratory laparotomy if the results of abdominal ultrasound were positive. A CT scan was performed for every BAT patient who was hemodynamically stable, either initially or after fluid resuscitation, to detect the presence and extent of intra-abdominal injuries. CT scans were performed at a 5-mm interval with and without contrast (Iopamidol, Italy). Hemodynamically stable patients with BAT underwent laparoscopy for select indications, including suspected hollow viscus injuries (as indicated by clinical or radiological findings), suspected diaphragm injuries, failed NOM for liver injuries with or without TAE, failed NOM for spleen injuries with or without TAE, isolated intra-abdominal fluid accumulation of uncertain origin shown on CT films and clinical findings. Failed NOM for patients with solid organ injuries (liver or spleen) was defined as patients exhibiting peritonitis or requiring continuous blood transfusion in order to remain hemodynamically stable. Patients undergoing laparoscopy had to meet the criteria and be kept in a stable hemodynamic status under fluid resuscitation and had no contraindications of pneumoperitoneum (severe head injury or cardiopulmonary insufficiency). In addition, the attending surgeon had to think that laparoscopy was feasible. Ultimately, the decision to perform a laparoscopy rested with the individual, attending surgeon. In our institution, laparoscopy was used as a treatment option for patients with failed NOM for liver or spleen injuries in this time period because an interventional radiology service was not available on a 24-hour basis. All the attending surgeons who performed laparoscopies for BAT patients had experience in advanced laparoscopic procedures (such as splenectomy or colectomy) for elective operations and had used laparoscopy to treat patients with stab wounds since 2006. Patients underwent a laparotomy if they were pregnant, were not eligible for laparoscopy, had undergone failed NOM for kidney injuries, or if was deemed appropriate based on the surgeon’s clinical judgment. Diagnostic and therapeutic laparoscopies were performed using techniques we previously reported [9,13,15,18] and the technique reported by Carobbi et al. [17] These procedures were performed using either a totally laparoscopic or laparoscopically assisted technique.

Patients received postoperative care and follow-up after discharge by trauma surgeons in both groups. All the patients were followed up after discharge for at least 6 months to detect long-term complications such as ventral hernias.

### Statistical analysis

Data are presented as the mean (standard deviation: SD), and comparisons between groups were performed using an independent 2-sample t test for continuous variables. Chi-squared/Fisher’s exact tests were used for categorical variables, and these data are presented as the count (percentage). Chi-squared tests were performed with Yates' correction for continuity. Except Fisher’s exact tests, all the other statistical assessments were two-tailed. We have reported values that are double the one-tailed exact probability when using Fisher’s exact tests in order to make the test results symmetrical. Statistical tests were evaluated at the .05 level for determining significant differences and were performed by using SPSS 15.0 statistics software (SPSS Inc., Chicago, IL, USA)

## Results

**Fig 1:**
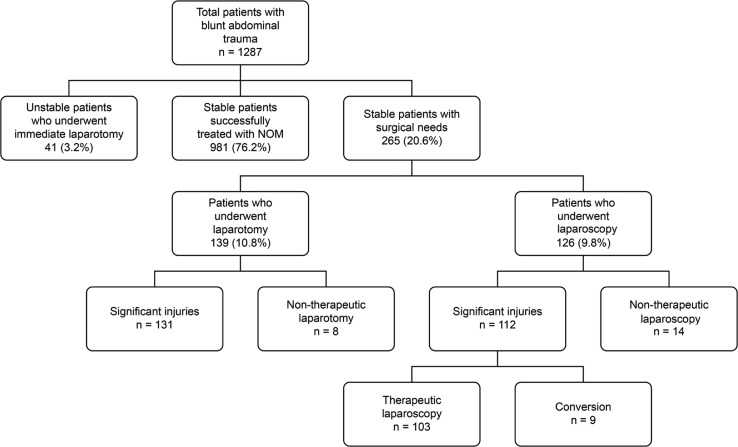
Patient summary flow chart.

There were 1287 BAT patients admitted to the trauma center over the 10-year period of this study. The patient flow chart is summarized in Fig 1. Forty-one patients underwent an exploratory laparotomy directly for persistent hypotension that was unresponsive to fluid resuscitation. They were severely injured, with a mean ISS of 39.0 (SD: 11.3) and a mortality rate of 58.5% (24/41). For patients who were hemodynamically stable either initially or after fluid resuscitation, 981 were successfully treated with NOM. The patients treated with NOM had a mean ISS of 16.5 (SD: 10.0), a rate of associated injuries of 73.7% (723/981), a rate of adjunctive TAE of 13.9% (137/981), a mean ICU stay of 1.8 (SD: 4.8) days, a mean hospital stay of 9.3 (SD: 8.2) days, and a mortality rate of 2.2% (22/981).

Of the 265 hemodynamically stable patients undergoing a surgical intervention for abdominal injuries, 139 were in group A, and 126 were in group B. The indications for surgery are shown in Table 1. The demographic data and treatment outcomes of patients are shown in Table 2. Patients in the 2 groups were similar in terms of sex, age, vital signs in the ED, associated injuries, TAE prior to surgery, and significant injuries requiring therapeutic interventions (all, *P* > .05). The patients in group A were more severely injured than the patients in group B; group A had a higher ISS (23.3 vs. 18.9, *P* < .001) and a higher frequency of associated TBIs (25.2% vs. 14.3%, *P* = .039). Although lower hemoglobin levels in the ED (12.5 vs. 13.1, *P* = .022) and higher abdominal AIS scores (3.7 vs. 3.3, *P* < .001) were observed for patients in group A, the differences were not clinically significant. Patients in the 2 groups had similar perioperative and postoperative outcomes in terms of operation times, blood loss, blood transfusion requirements, mortality, and complications (all, *P* > .05). However, the laparoscopy-based approach resulted in no non-therapeutic laparotomies for patients in group B. Patients in group A had significantly longer hospital stays (19.4 vs. 12.1 days, *P* < .001) and longer ICU stays (6.6 vs. 3.3 days, *P* < .001). Significant injuries and operative procedures for both groups are shown in Table 3. Approximately two thirds of the procedures for patients in group B required advanced laparoscopic techniques, and one third required advanced instruments.

**Table 1:** Indications for surgery.

	Laparotomy (*n* = 139)	Laparoscopy (*n* = 126)
Suspected hollow viscus injuries	17	23
Suspected diaphragm injuries	4	7
Failed NOM for liver injuries	35	22
Failed NOM for spleen injuries	50	40
Failed NOM for kidney injuries	3	0
Isolated intra-abdominal fluid accumulation of uncertain origin shown on CT films and clinical findings	30	34

CT, computed tomography; NOM, non-operative management

**Table 2:** Patient demographic and clinical characteristics.

	Laparotomy (*n* = 139)	Laparoscopy (*n* = 126)	*P* value
Sex (M:F)	99:40	80:46	0.226^‡^
Age (years)	35.2 (16.2)	38.5 (18.0)	0.117^†^
Initial SBP (mmHg)	128.6 (22.9)	126.6 (26.2)	0.490^†^
Initial HR (bpm)	95.5 (18.7)	91.4 (18.7)	0.075^†^
AIS abdomen	3.7 (0.7)	3.3 (0.6)	< 0.001^†^
ISS score	23.3 (9.9)	18.9 (8.5)	< 0.001^†^
Hemoglobin (mg/dl)	12.5 (2.6)	13.1 (2.0	0.022^†^
Associated injuries, n (%)	91 (65.5)	81 (64.3)	0.942^‡^
Associated TBIs, n (%)	35 (25.2)	18 (14.3)	0.039^‡^
TAE prior to surgery, n (%)	5 (3.6)	4(3.2)	1.000^§^
Significant injuries, n (%)	131(94.2)	112(88.9)	0.175^‡^
Operative procedures, n (%)			NT
Positive, non-therapeutic	8 (5.8)	14 (11.1)	
Positive, therapeutic	131 (94.2)	103 (81.8)	
Positive, conversion	NA	9 (7.1)	
Conversion to laparotomy, n (%)	NA	9 (7.1)	
Non-therapeutic laparotomy, n (%)	8 (5.8)	0 (0.0)	0.010^§^
Operation time (minutes)	125.4 (43.4)	131.4 (48.3)	0.293^†^
Blood loss (ml)	1515.3 (1410.1)	1187.2 (1494.2)	0.067^†^
Blood transfusion requirement, n (%)	84 (60.4)	67(53.2)	0.286^‡^
Length of hospital stay (days)	19.4 (12.2)	12.1 (7.2)	< 0.001^†^
Length of ICU stay (days)	6.6 (7.7)	3.3 (4.9)	< 0.001^†^
Mortality (%)	5 (3.6)	1 (0.8)	0.262^§^
Complications (%)	19 (13.7)	9 (7.1)	0.127^‡^
Missed injuries requiring reoperation	1 (0.7)	1 (0.8)	1.000^§^
Wound infection (%)	12 (8.6)	5 (4.0)	0.195^‡^
Intra-abdominal abscess (%)	7 (5.0)	3 (2.4)	0.420^§^
Long-term complications (%)	9 (6.5)	3(2.4)	0.192^‡^

SBP, systolic blood pressure; AIS, abbreviated injury scale; ISS, injury severity scores; ICU, intensive care unit; TBI, traumatic brain injuries; TAE, trans-arterial embolization; NT, no significance test conducted; NA, non-applicable.

Data are reported as the mean (standard deviation) or number (percentage).

^†^Student’s *t* tests

^‡^χ^2^ test

^§^Fisher’s exact test.

**Table 3:** Significant injuries and procedures.

Significantly injured organs/Procedures	Laparotomy (*n* = 131)	Laparoscopy (*n* = 112)
Mesentery and omentum	11	16
Ligation of bleeder and repair	4	10
Bowel resection and anastomosis	7	6
Stomach	3	2
Primary closure of stomach	3	2
Duodenum	2	0
Primary closure of duodenum	2	
Small bowel	16	28
Primary closure of small bowel	9	15
Bowel resection and anastomosis	7	13
Colon and rectum	13	9
Primary closure of colon	4	1
Repair or resection and ostomy	9	8
Liver	38	25
Hepatorrhaphy	30	22
Hepatectomy	8	3
Gall bladder	5	2
Cholecystectomy	5	2
Pancreas	9	6
Hemostasis and drainage	4	4
Distal pancreatectomy	5	2
Spleen	53	42
Splenorrhaphy	4	36
Splenectomy	49	6
Kidney	5	0
Nephrectomy	4	
Nephrorrhaphy	1	
Ovary	0	1
Oophorectomy		1
Urinary bladder	5	7
Primary closure of urinary bladder	5	7
Diaphragm	5	3
Repair of diaphragm	5	3
Miscellaneous	3	4
Repair of traumatic abdominal wall hernia	3	4

The demographic data and treatment outcomes of patients operated on following failed NOM for solid organ injuries (liver or spleen) are shown in Table 4. The ratio of patients with solid organ injuries to all patients was similar in both groups (61.2%, 85/139 vs. 63.6%, 66/126, *P =* .188). There were more patients with spleen injuries in both groups (62.4%, 53/85, vs. 63.6%, 42/66, *P =* 1.000). The patients with solid organ injuries in group A were also more severely injured than the patients with solid organ injuries in group B in that they had higher ISSs (25.7 vs. 21.8, *P* < .001). However, the incidences of associated injuries (67.1% vs. 60.6%, *P* = .516) and associated TBIs (28.2% vs. 19.7%, *P* = .308) were not different. The laparoscopy-based approach increased the spleen salvage rate (7.5%, 4/53 vs. 85.7%, 36/42, *P* < .001). However, a longer operation time (113.5 vs. 135.8 min, *P* = .001) was noted for patients with solid organ injuries in group B. Patients with solid organ injuries in both groups had similar perioperative and postoperative outcomes in terms of blood loss, blood transfusion requirements, mortality, and complications (all, *P* > .05). As shown in Table 2 and Table 4, patients with solid organ injuries had higher levels of blood loss when compared with all patients (group A:1971 vs. 1515 ml; group B: 1888 vs. 1187 ml). Patients with solid organ injuries in group B also had a significantly shorter hospital stays (17.7 vs. 12.2 days, *P* < .001) and shorter ICU stays (7.9 vs. 4.2 days, *P* = .001).

**Table 4:** Patient demographic and clinical characteristics for liver and spleen injuries.

	Laparotomy (*n* = 85)	Laparoscopy (*n* = 66)	*P* value
Ratio relative to all patients (%)	61.2	52.4	0.188^‡^
Sex (M:F)	60:25	37:29	0.094^‡^
Age (years)	35.1 (16.0)	35.9 (16.3)	0.754^†^
Initial SBP (mmHg)	129.5 (24.2)	128.0 (27.6)	0.716^†^
Initial HR (bpm)	93.9 (19.3)	95.2 (18.0)	0.681^†^
AIS abdomen	3.8 (0.7)	3.5 (0.6)	0.003^†^
ISS score	25.7 (10.8)	21.8 (8.6)	< 0.001^†^
Hemoglobin (mg/dl)	12.2 (2.4)	12.6 (2.0)	0.240^†^
Associated injuries, n (%)	57 (67.1)	40 (60.6)	0.516^‡^
Associated TBIs, n (%)	24 (28.2)	13 (19.7)	0.308^‡^
TAE prior to surgery, n (%)	5 (5.9)	4(6.1)	1.000^§^
Liver: spleen	32:53	24:42	1.000^‡^
Spleen salvage, n (%)	4/53 (7.5)	36/42 (85.7)	< 0.001^‡^
Operation time (minutes)	113.5 (36.7)	135.8 (46.3)	0.001^†^^.^
Blood loss (ml)	1971.2 (1471.6)	1888.8 (1708.9)	0.751^†^
Blood transfusion requirement, n (%)	71 (83.5)	51 (77.3)	0.447^‡^
Length of hospital stay (days)	17.7 (11.1)	12.2 (6.6)	< 0.001^†^
Length of ICU stay (days)	7.9 (7.8)	4.2 (4.9)	0.001^†^
Mortality (%)	3 (3.5)	1 (1.5)	0.822^§^
Complications (%)	8 (9.4)	3 (4.5)	0.412^§^

*SBP, systolic blood pressure; AIS, abbreviated injury scale; ISS, injury severity scores; ICU, intensive care unit; TBI, traumatic brain injuries; TAE, trans-arterial embolization.

Data are reported as the mean (standard deviation) or number (percentage).

^†^Student’s *t* tests

^‡^χ^2^ test

^§^Fisher’s exact test.

### Laparotomy group

Of the 139 hemodynamically stable patients undergoing an exploratory laparotomy, the most common indication was patients who experienced failed NOM for spleen injuries (36.0%, 50/139), and the second most common indication was patients who experienced failed NOM for liver injuries (25.2%, 35/139). The sensitivity of laparotomy in group A was 99.2% (130/131), with 1 patient requiring reoperation for a missed rectal ischemic injury following the repair of small bowel perforations.

Of the 8 patients who underwent a non-therapeutic approach, 7 were operated on owing to isolated intra-abdominal fluid accumulation shown on CT films and 1 owing to a suspected diaphragm injury. These insignificant findings included 5 (62.5%) retroperitoneal hematomas, 1 (12.5%) non-bleeding liver laceration, and 2 (25.0%) non-expanding colonic hematomas. All of these patients had an uneventful recovery, so the specificity of laparotomy was 100.0% (8/8) in group A. As shown in Table 3, 131 patients (94.2%) patients underwent therapeutic procedures for a total of 163 intra-abdominal injuries. The procedures included 4 repairs of mesenteric or omental bleeding, 7 bowel resections and anastomoses for mesenteric injuries, 3 gastrorrhaphies, 2 duodenorrhaphies, 9 repairs of small bowel injuries, 7 bowel resections and anastomoses of small bowel injuries, 4 repairs of colon injuries, 9 resections and anastomoses or enterostomies of colon injuries, 30 hepatorrhaphies, 8 hepatectomies, 5 cholecystectomies, 4 drainages of pancreatic injuries, 5 distal pancreatectomies, 4 splenorrhaphies, 49 splenectomies, 5 cystorrhaphies, 1 nephrorrhaphy, 4 nephrectomies, 5 repairs of diaphragm injuries, and 3 repairs of traumatic abdominal hernias.

The mortality rate was 3.6% (5/139) for patients in group A. Two patients died from an intractable TBI, 2 from associated chest injuries, and 1 from intra-abdominal injuries. A 73-year-old male patient died on hospital day 5 due to profound sepsis after an ileostomy for transverse colon perforations on hospital day 2. Nineteen (13.7%) patients had postoperative complications in group A. There were 7 intra-abdominal abscesses (2.3%), 12 wound infections (8.6%), 5 cases of postoperative ileus (3.6%), and 4 ventral hernias (2.9%), which occurred at 2–6 months postoperatively.

### Laparoscopy group

The most common indication for laparoscopy in group B was failed NOM for spleen injuries (31.7%, 40/126), and the second most common indication was patients with isolated fluid accumulation shown on CT films and clinical findings (27.0%, 34/126). The sensitivity of diagnostic laparoscopy in group B was 99.1% (111/112), with 1 patient requiring a reoperation for a missed urinary bladder perforation after a diagnostic laparoscopy for a pelvic hematoma.

Of the 126 patients in group B, 14 (11.1%) underwent non-therapeutic laparoscopy. Eleven (78.6%) of them underwent non-therapeutic laparoscopy based on isolated intra-abdominal fluid accumulation shown on CT films. All 14 patients had an uneventful recovery, so the specificity of laparoscopy was 100.0% (14/14) in group B. No non-therapeutic laparotomies were performed. For the 112 (88.9%) patients, with a total 145 significant intra-abdominal injuries identified by laparoscopy, the success rate of therapeutic laparoscopy was 92.0% (103/112), with 9 patients requiring a conversion to laparotomy. As shown in Table 3, therapeutic laparoscopic procedures performed for patients in group B included 10 repairs of mesenteric or omental bleeding, 6 bowel resections and anastomoses for mesenteric injuries, 2 gastrorrhaphies, 15 repairs of small bowel injuries, 13 bowel resections and anastomoses of small bowel injuries, 1 repair of colon injuries, 8 resections and anastomoses or enterostomies of colon injuries, 22 hepatorrhaphies, 3 hepatectomies, 2 cholecystectomies, 4 drainages of pancreatic injuries, 2 distal pancreatectomies, 36 splenorrhaphies, 6 splenectomies, 7 cystorrhaphies, 1 oophorectomy, 3 repairs of diaphragm injuries, and 4 repairs of traumatic abdominal hernias.

Of the 9 patients requiring a conversion to laparotomy, 5 underwent conversions based on the surgeon’s experience, and the other 4 were converted after they became hemodynamically unstable during the laparoscopic procedures. The procedures in cases of conversion to laparotomy included 4 splenectomies, 2 bowel resections and anastomoses, 1 liver resection, and 2 liver resections and distal pancreatectomies. Overall, the laparoscopy-based approach avoided laparotomy in 92.1% (116/126) of BAT patients in group B who were suspected of needing surgery.

There was 1 death in group B. A 57-year-old male who underwent a conversion to laparotomy for blunt liver and pancreatic injuries died on hospital day 24 due to multiple organ failure. Nine patients in group B developed postoperative complications, with 3 intra-abdominal abscesses (2.4%), 5 wound infections (4.0%), 1 case of postoperative ileus (0.8%), and 3 ventral hernias (2.4%), which developed at 2–3 months postoperatively.

## Discussion

This study compared the outcomes of 126 hemodynamically stable BAT patients with surgical needs treated by laparoscopy with the outcomes of 139 patients treated by laparotomy in the same time period. Our previous studies have suggested the role laparoscopy can play for select BAT patients, except for those with spleen injuries [13,15]. Since then, the application of laparoscopy for BAT patients had gained more traction in expanded clinical scenarios, including spleen injuries, for use by emergency surgeons in our hospital. The current study was designed to validate the feasibility and effectiveness of laparoscopy relative to laparotomy for treating BAT patients by comparing the results of a single surgical team adopting the same treatment algorithm with expanded indications over a 10-year period. With accumulated the experience of attending surgeons in advanced laparoscopic procedures and laparoscopy for treating abdominal trauma, this 10-year study showed that laparoscopy can be effectively used to diagnose and treat injuries in select patients with BAT, a relatively more complex injury pattern than that of stab wounds. The present study further illustrated that when performed by experienced surgeons, diagnostic and therapeutic laparoscopy was beneficial for hemodynamically stable BAT patients in select scenarios, including patients with suspected hollow viscus injuries or suspected diaphragm injuries, those undergoing failed NOM for liver or spleen injuries, or those with isolated intra-abdominal fluid and clinical findings. The laparoscopy-based approach avoided the need of laparotomy and had similar perioperative and postoperative outcomes. Compared to our previous studies [13,15], we believe that the current results are more informative for use as a references for daily practice by emergency surgeons regarding the value of laparoscopy in treating patients with BAT. This opinion is based on the fact that in the present study, more patients were recruited over a longer study period, the procedures were performed by surgeons on a single surgical team and using the same treatment algorithm, the indications of laparoscopy were expanded, and the importance of the experience levels of surgeons was addressed.

The indications for a surgical intervention were similar in both groups, except for patients who underwent failed NOM for renal injuries. Patients in group A were more severely injured than the patients in group B as shown by a higher ISSs and a higher frequency of associated TBIs. The difference in the injury severities between the 2 groups might be due to difference in clinical judgment regarding the uses of laparoscopy between individual surgeons. The reason why more patients had TBIs in the laparotomy group was that we excluded patients with severe head injuries from undergoing laparoscopy. There are safety concerns arising from the concepts proposed by Josephs et al., who have shown that carbon dioxide pneumoperitoneum causes a significant increase in intracranial pressure in a porcine model of head injury [19]. Because the disease severity of patients in group B was lower than for patients in group A, similar or better surgical outcomes, including operation time, blood loss, length of ICU stay, length of hospital stay, mortality, and complications, could be anticipated. The sensitivity and specificity of diagnostic laparoscopy was as high as 99.1% and 100.0% in this study, implying the value of laparoscopy to avoid non-therapeutic laparotomy for BAT patients. In addition, the laparoscopy-based approach had a high success rate (92.0%) of therapeutic interventions for patients with significant injuries in group B. The results of this study confirm that laparoscopy can play a major role for select BAT patients in a stable hemodynamic status, both for diagnosis and treatment, which is similar to findings in our previous reports for stab wounds [9]. All the attending surgeons who performed laparoscopies on BAT patients in this study had gained experience in advanced laparoscopy and had treated patients with stab wounds using laparoscopy during the 3 years prior to the period of the current study. These results validate our hypothesis that although performing laparoscopy for patients with BAT is more technically challenging, good outcomes can be achieved if the surgeons have adequate experience in advanced laparoscopic procedures and using in laparoscopy to treat PAT.

Laparoscopic techniques have been widely used in many types of general surgery, but confidence in using laparoscopy to manage injured patients has not been well-established [20]. Other than the diagnostic value of reducing non-therapeutic laparotomy rates for patients with PAT, the benefits of MIS for injured patients cannot be highlighted until its therapeutic role is established. On the basis of accumulated experience in using laparoscopy to treat traumatic injuries, a number of studies have reported the use of therapeutic laparoscopy for suitable patients [9,13,20–24], but the success rates have varied in these studies. In a recent report analyzing more than 2.5 million trauma center visits from 2007 to 2010 in the United States, 4755 patients underwent diagnostic laparoscopy for abdominal trauma at 467 trauma centers; 68.7% of these patients sustained PAT, and only 19.3% of them underwent a therapeutic intervention. Although the success rate of therapeutic laparoscopy for trauma patients was less than 20% in the study, patients undergoing laparoscopic surgery were proven to have a significantly shorter stays than those who underwent an open procedure [22]. A systematic review by O’Malley et al. [25], including 51 studies of patients with PAT, demonstrated an overall 13.8% success rate for therapeutic laparoscopy for 1129 patients with significant injuries identified by 2569 diagnostic laparoscopies. In the present study, the success rate of therapeutic laparoscopy was 92.0% for patients in group B, which implies that BAT patients can also benefit from laparoscopic management in select scenarios if attending surgeons have adequate experience.

The present study emphasizes the value of therapeutic laparoscopy for patients with significant intra-abdominal injuries. The keys to performing therapeutic laparoscopy successfully include avoiding missed injuries and maximizing therapeutic interventions. In this study, the rate of missed injuries in the laparoscopy group was minimized to 0.9% by using a standardized examination method, compared with an average missed injury rate of 3.2% [25]. The importance of using a standardized examination method in laparoscopy for trauma has been reported before [8,13] and was further confirmed in the current study.

The keys to maximizing the therapeutic potential of laparoscopy for patients with abdominal trauma include the incorporation of new therapeutic procedures, the availability of advanced instruments, surgeons’ experience in using laparoscopy to treat traumatic injuries, and determining an optimal threshold of conversion to laparotomy. Although laparoscopy has been effectively used for treating a variety of injuries, the spectrum could be expanded by adopting newly developed techniques. The adoption of a new therapeutic procedure is related to surgeon experience and institution-specific facilities. In this study, surgeons managed patients with spleen or liver injuries who experienced failed NOM with the laparoscopic “sandwich repair technique” we reported previously [18]. Although TAE has been regarded as an effective adjunctive method for liver [12] and spleen [11] injuries, “the sandwich repair" technique was developed because an interventional radiology service was not available on a 24-hour basis in our institution. Furthermore, laparoscopy was used as a salvage procedure for liver or spleen injuries prior to laparotomy if TAE had failed. For patients who experienced failed NOM for liver or spleen injuries, the laparoscopic repair technique provided patients favorable outcomes in terms of increasing spleen salvage (7.5% vs. 85.7%, *P*< .001). Some might argue that patients with solid organ injuries should be managed by laparotomy for quicker bleeding control because predominant blood loss, up to 1900 ml, was noted in patients in group B. However, the similar amounts of blood loss (2000 ml) was noted in patients with solid organ injuries in group A, and the clinical outcomes of patients in the 2 groups were not different. These findings imply that this amount of blood loss can be tolerated by patients when performing laparoscopic techniques to treat solid organ injuries. Based on these results, laparoscopy could be regarded as feasible and safe for patients with spleen or liver injuries.

In terms of advanced instruments, we recommended the use of a Harmonic Scalpel (Johnson &Johnson, Cincinnati, OH, USA) for bleeding mesenteries or the omentum and a LigaSure (Covidien, Mansfield, MA, USA) for torn vessels at the splenic hilum to facilitate therapeutic techniques. In the current study, about one third of the patients in group B underwent a laparoscopic procedure successfully with the aid of advanced instruments. The value of using advanced instruments to facilitate hemostasis has been shown in our previous series [18] and was proven again in this study.

Another important factor relating to a successful therapeutic laparoscopy is the surgeon’s experience with using laparoscopy for traumatic injuries. It is technically demanding to treat complex injuries using a laparoscopy-based approach. The success rate of therapeutic laparoscopy for treating more complex injuries resulting from BAT is related to the surgeon’s experience. Advanced laparoscopic techniques, such as intra-corporeal suturing, offer the opportunity to increase the success rate of therapeutic laparoscopy in the treatment of complex injuries. Our previous study suggested that a surgeon can be regard as experienced if he or she has performed 5 laparoscopic procedures for stab wounds before treating patients with BAT [13]. In this study, the experience of the surgeons should be regarded as adequate because all of them had experience in laparoscopic splenectomy or colectomy for elective operations and had been managing patients with stab wounds for 3 years prior to the study period. In addition to gaining experience in the use of laparoscopy for treating stab wounds, we further emphasize the value of performing advanced laparoscopic procedures as part of elective operations because surgeons can gain the laparoscopic skills required for treating BAT patients.

An optimal threshold for conversion to laparotomy is based on whether the surgical team is comfortable with performing the required procedures and if the patient remains hemodynamically stable throughout the procedures [22]. Most reported series have emphasized the importance of a stable hemodynamic status in MIS for traumatic injuries [9,13,21]. It is advised that laparoscopy be used cautiously in patients with severe BAT. In this study, we excluded patients with persistent hypotension unresponsive to fluid resuscitation because emergency laparotomy can be life-saving for hemodynamically unstable patients. In addition, no attempt to repair complex injuries by laparoscopy should be considered when a patient’s hemodynamic status has deteriorated. It is crucial to emphasize that BAT patients are able to benefit from MIS only if they remain hemodynamically stable.

This was a retrospective study designed to compare the effectiveness of a surgical procedure to a standard method in a single institution with limited patient numbers, and there are some limitations to this report. First, the impact of selection bias in patient selection due to individual surgeon’s preferences could not be evaluated. Second, injury severities of the patients in the 2 compared groups could not be adjusted. Third, the impact of associated injuries on the perioperative outcomes of patients in the 2 groups is difficult to estimate. Finally, this study was done at a medical center where trauma surgeons are skilled in advanced laparoscopic procedures; whether similar results can be reproduced at other medical centers deserves further study.

## Conclusions

When performed by experienced surgeons, laparoscopy is a feasible and safe tool for the diagnosis and treatment of hemodynamically stable BAT patients for select surgical scenarios. These include suspected hollow viscus injuries, suspected diaphragm injuries, failed NOM for liver or spleen injuries, or patients with isolated intra-abdominal fluid and clinical findings. Laparoscopy can be used to avoid a non-therapeutic laparotomy and to perform therapeutic interventions for these patients.

## Supporting information

S1 File The demographic and clinical data of patients in both groups. (SAV)

S2 File The demographic and clinical data of patients with spleen or liver injuries in both groups. (SAV)
